# Simulation as an educational tool to teach emergency medicine residents about unconscious bias

**DOI:** 10.1007/s43678-024-00679-3

**Published:** 2024-03-26

**Authors:** Nadia Primiani, Lara Murphy, Stephanie Dephoure, Cameron Thompson, Carly Ng

**Affiliations:** 1 Department of Family and Community Medicine, Sinai Health System, Michael Garron Hospital, University of Toronto, Toronto, ON Canada; 2 Temerty Faculty of Medicine, University of Toronto, Toronto, ON Canada; 3 Schwartz/Reisman Emergency Medicine Institute, Sinai Health System, Toronto, ON Canada; 4 Dalla Lana School of Public Health, University of Toronto, Toronto, ON Canada; 5 Department of Family and Community Medicine, University Health Network, University of Toronto, Toronto, ON Canada

**Keywords:** EDIIA, Unconscious bias, Simulation, Emergency medicine, EDIIA, Préjugés inconscients, Simulation, Médecine d’urgence

## Abstract

Medical training embraces simulation-based education. One important topic that has recently been added to the simulation curriculum at the University of Toronto is unconscious bias. This educational innovation project evaluates a simulation that could be used as a novel instructional design strategy to teach unconscious bias. The simulation involved two resuscitation scenarios with a similar clinical trajectory. Each resuscitation was led by standardized physicians of different binary genders, followed by a debrief with highly trained facilitators. Tangible educational takeaways on team dynamic variation between different gendered team leaders were discussed following the simulation, highlighting its benefit to resident education on bias awareness and response. Limitations include inevitable unconscious bias in facilitators and unequal gender representation in the learner participants, which may impact simulation effectiveness. The findings support translating this simulation to other forms of bias education in future simulation development.

**Supplementary Information:**

The online version contains supplementary material available at 10.1007/s43678-024-00679-3.

## Résumé

La formation médicale englobe l’éducation basée sur la simulation. Un sujet important qui a récemment été ajouté au programme de simulation de l’Université de Toronto est celui des préjugés inconscients. Ce projet d’innovation éducative évalue une simulation qui pourrait être utilisée comme une nouvelle stratégie de conception pédagogique pour enseigner les préjugés inconscients. La simulation a impliqué deux scénarios de réanimation avec une trajectoire clinique similaire. Chaque réanimation a été menée par des médecins standardisés de sexe binaire différent, suivis d’un débriefing avec des facilitateurs hautement qualifiés. Après la simulation, on a discuté de points concrets à retenir sur la variation de la dynamique d’équipe entre les différents chefs d’équipe selon le sexe, en soulignant les avantages pour l’éducation des résidents sur la sensibilisation aux préjugés et la réponse. Les limites comprennent des préjugés inconscients inévitables chez les animateurs et une représentation inégale des sexes chez les apprenants participants, ce qui peut avoir une incidence sur l’efficacité de la simulation. Les résultats appuient la traduction de cette simulation à d’autres formes de formation sur les préjugés dans le développement futur de la simulation.

## Background

Microaggressions towards women and those underrepresented in medicine are the most reported form of abuse by resident physicians. Experiencing microaggressions has been linked to the development of anxiety, depression, and hypertension [1, 2], and contributes to increased burnout [3, 4]. Thus, it is paramount to train learners to better recognize and address this bias within clinical settings. The Canadian Association of Emergency Physicians (CAEP) encourages educational initiatives to address and mitigate gender inequity and its intersections with other forms of marginalization [5]. Simulation training has been shown to improve knowledge, clinical skills, and behaviours [6], while improving downstream effects on patient care [7]. To date, a simulation case focused on unconscious binary gender biases while leading resuscitations has not been developed.

Duchesne et al. published a pilot study examining simulation and reflective teaching on gender-based microaggressions to foster allyship and practice responses to microaggressions [8]. This supported simulation as a potential modality for Equity, Diversity, Indigeneity, Inclusion and Accessibility (EDIIA) curriculum innovations.

Gender exists on a spectrum. When we refer to “women”, we include all who identify as women or have had experience as women and/or the feminine side of the gender spectrum. The opposite is true when we refer to “men” [9].

### Purpose and rationale

The purpose of this work was to develop and describe a simulation innovation that could be used as a novel instructional design strategy for education on recognizing unconscious gender biases. In doing so, learners could connect their individual biases to systemic ones. Residents participated in a simulation with embedded errors run by leads of different genders, followed by a structured debrief.

### Description of the innovation

All residents enrolled in the University of Toronto Canadian College of Family Physicians Emergency Medicine (CCFP(EM)) program participated in this educational innovation as part of a simulation curriculum that is run longitudinally to their clinical rotations.

A simulation was written by a faculty member with an interest and education in EDIIA. The overall goal was to reveal and challenge unconscious gender biases within residents. This was developed with the principle of transformative learning theory (TLT), whereby the learners were placed in a situation where they may have concerns with directions given by the team lead. Objectives were recognizing how their interactions differed between team leads (TL) of different genders, and how this might be extrapolated to other experiences. This was paired with discussing strategies to minimize the impact of bias on future team interactions.

The cases were written to be identical, with scripted and timed errors. They were run consecutively (Appendix A). The first simulation was led by a cis-female TL and the second case by a cis-male TL of presumed similar ethnic background. Both team leads were trained, embedded, standardized participants who were not part of the research team. Prior to the simulations, a pre-brief was held to discuss psychological safety with an emphasis on safeR spaces and informing learners of a social worker that they could access. A debrief of both simulations was held immediately following the conclusion of the second simulation. The facilitators were experienced simulation debrief instructors with expertise in the impacts of gender bias and microaggressions. During the debriefs, the participants and TLs both shared how they responded to team dynamics during the simulation.

A total of six residents, five of whom identified as cis-male and one as cis-female, participated in the simulations (Table 1). Participants were organized into two simulation groups, which became their subsequent focus groups.

**Table 1:** Participant* quotations regarding the simulation (*to maintain participant anonymity, some of the gender of participants is not included)

Participant	Quotes
Female lead	It was very, for me, very obvious as a leader, the difference…in my case, there was a lot of pushback… other people took over and started saying we’re going to do this, and this, and this… if you are familiar with the case before, and you are not comfortable with intubation the first time, you would think that there would be more of a push back in the second case … but it was interesting to see how there was a lot less.
Resident	I feel like I can relate to that being also a small [gender], and I think it can, you know, it can be hard I think sometimes to say, like, “I’m the leader”. … I think when that happens it can be harder to take it back despite people, like, trying to almost give it back to you
Resident	I mean we talked about just your presence in the room, the way you stand, the way you speak, can make a huge difference in the way sort of people respond to you, and how sort of the code or resus goes
Resident	Even when you’re running a code…I find that when I have a female staff that I’m working with and I’m running a code, the team will give me more attention and let me be the leader more so than if I was running the code with a male staff where the team would defer a little bit more to the male staff and not give me the full attention. So, there’s certainly biases there and it’s easy to pick up on, and often it can be implicit, right, because as a male you benefit from it, so it’s hard to, like, kind of call these things out, but it’s certainly real
Resident	I know we didn’t talk about privilege, but I think when we talk about biases we have to recognize our privilege as the common point to everything. So, I think, you know, recognizing our own privilege and how you can be an ally in certain situations…
Resident	For me, it’s being aware of how my position might actually lead to people listening or following orders when they don’t think it’s safe to do so. That’s the big takeaway, I hadn’t really thought of that at all
Resident	I feel like we’re going into leadership roles in three months’ time where we’re going to have learners, where we’ll be with other staff …– being males, we will inherently be given that different level of respect based on our sex or based on some other characteristic, whether obvious or not obvious about this as well, and I think, like, what you’re saying is we just kind of call it out or at least go into all of these situations having some understanding of this will happen, so try and be attuned to it

To replicate the simulation, we recommend selecting debriefing instructors who have significant training in unconscious bias education.

### Participant feedback

Both quantitative and qualitative feedback were sought from participants in the form of online surveys and transcribed focus groups. Residents did become increasingly aware of their own experience of biases and microaggressions following the simulation. Qualitative analysis of the debriefs found that with the cis-female TL, residents provided greater pushback toward her instructions and even took over the case direction when they disagreed with her management directions. Contrastingly, the cis-male lead felt supported by his team and participants were less forthcoming with criticism of his directions. Participants were surprised at the difference in treatment of the cis-female TL during the debrief. This “disorienting” experience led to critical reflection, dialogue, and a commitment to action.

Having the embedded physician participants join in the structured debrief, allowed for them to discuss how they experienced the biases in the simulation. This led to fruitful discussion around the unintended consequences of the team’s actions. Furthermore, they were able to discuss these experiences in the context of previous lived experiences of bias in their own careers. Please see example debrief questions in Appendix A.

## Discussion

We demonstrated that simulation-based medical education can be used to increase learner awareness of their unconscious biases and how these connect to systemic bias. Further, this design encouraged reflection on strategies to mitigate and address the impact of bias in their teams. Eliciting such differences in a controlled setting can help counteract participant perceptions that unconscious bias is only perpetrated by others, and not within themselves. Demonstrating bias can thus challenge cognitive distancing from fault, allowing for better recognition of how biases may similarly impact their clinical interactions.

While this simulation focused on unconscious bias, the debriefing also allowed for comprehensive self-reflection around communication, team dynamics, and microaggressions, allowing learners the opportunity to deconstruct events and learn from errors to improve future practice. This form of learning theory was taken from TLT, which can instigate social change by promoting critical engagement and “fostering the creation of more equitable conditions” [10]. This can allow learners to move from bias recognition to tangible transformation and action [10].

Strategies discussed to counteract biases in clinical environments primarily surrounded allyship with female residents and staff. Other strategies included addressing bias when recognized and proactively creating an environment conducive to constructive feedback. Lastly, this simulation led to learners reflecting on the need for improved communication skills in a resuscitation environment, including the use of CUS words (I’m concerned, I’m uncomfortable, this is a safety issue) [10], which have been well studied. Strategies for supporting team members experiencing bias included checking in following the event that subjected them to bias and validating their experiences.

### Limitations

There are several limitations. The simulation results may not reflect lasting changes in behaviour or the long-term impact of simulations for unconscious bias learning in medical education.

This simulation was run with an unequal gender representation both in learners and instructors. Further research should examine how the composition of learner/instructor gender, and respective racial identities may influence learning effectiveness, debriefing styles, and learner engagement. As with all EDIIA work, we acknowledge that we have unconscious biases, and these may have affected the session [8].

Running the same case twice back-to-back led to some confounding from learners, changing their behaviour due to repetition. Future cases could be run with different diagnostic dilemmas and similar scripted mistakes.

Further, the TLs perception of the group behaviours may have been influenced by their knowledge of the study objective. This may have impacted the outcome of the simulation and the perspectives shared during the debrief. However, selecting external TLs knowledgeable of the scenario was intended to prevent personal biases from residents’ experiences working with each other. We also recognize that focusing on binary genders does not address intersectionality and the breadth of the gender spectrum [8].

## Summary

Simulation can be used to effectively teach about gender-based unconscious bias. Our innovation employed transformative learning theory, and debriefing allowed for self-reflection, group learning and strategies for behaviour changes. This further supports that simulation cases developed around unconscious bias training should ideally exist alongside a dedicated curriculum founded on EDIIA principles.

For a list of definitions, please see Appendix B.

### Supplementary Information

Below is the link to the electronic supplementary material.

Supplementary file1 (DOCX 6234 KB)

Supplementary file2 (DOCX 17 KB)

## Data Availability

The data that support the findings of this study are available from the corresponding author, NP, upon reasonable request.
