# Supplementary material for: Simulation as an educational tool to teach emergency medicine residents about unconscious bias
Source: CJEM. 2024 Mar 26;26(6):395–8. doi: 10.1007/s43678-024-00679-3 (PMC11189339; doi:10.1007/s43678-024-00679-3)
Supplement: Supplementary file 1 — Supplementary file1 (DOCX 6234 KB) [file 43678_2024_679_MOESM1_ESM.docx]

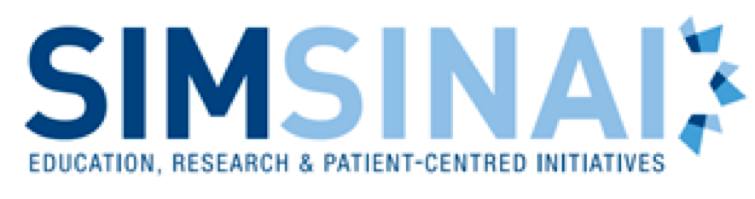


**UNCONSCIOUS BIAS**

**Title: Unconscious Bias**

**Date:** November 13, 2021

**Author:**

**Learning Objectives:**

At the end of this simulation, the learners will be able to:

1. Demonstrate effective teamwork and communication managing acute respiratory failure
2. Recognize gender differences in leading resuscitations
3. Acknowledge their own unconscious bias and microaggressions regarding gender
4. Develop tools to manage bias and microaggressions within the workforce

**Target Audience:** Emergency Medicine Fellows (CFPC(EM))

**Critical Action List (medical management):**

1. Recognize acute respiratory failure
2. Clearly articulate to the team the differential diagnoses of respiratory failure
3. Early recognition of heart failure using history, physical, POCUS and ECG findings
4. Quick transition to vasopressor support

**Case Overview:**

Kelli is a 73-year-old patient who has presented to the emergency room with acute shortness of breath and cough over the last few days. They have a history of COPD, CHF, hypertension and hyperlipidemia. On arrival, they are using accessory muscles and breathing at 32 breaths per minute. The vitals are a heart rate of 110 (irregular), BP 165/ 95, temp 37.5, and oxygen saturation 89% on 4L nasal prongs. The learners will be forced to approach this diagnostic dilemma with history, physical, POCUS and investigations to determine that they are suffering from acute heart failure.

**Pre-brief:** Ensure discussions on psychological safety, emphasis on safeR spaces and informing learners of an available social worker for them to access. The learners were told that the standardized leads were learners from other residency programs joining to get more practice with being team leads. Make it explicit that both scenarios will be identical, so each TL not involved will be away from watching the simulation unfold.

**Scenario prompt:**  Kelli is a 73-year-old patient who you’ve been asked to see in resus with acute shortness of breath and cough over the last few days. They have a history of COPD, CHF, hypertension and hyperlipidemia. They have been brought into the ED via EMS who have started 4L nasal prongs 100% O2, and sats 90s. They appear unwell and are using accessory muscles.

P 110bpm (a.fib) BP 165/95 RR 32 89% on 4L NP T 37.5C tympanic BG 5.0 mmol/L

**Patient's SAMPLE history:**

3-4 word dyspnea.

“feeling more shortness of breath over the past few days” “significantly worsened today after lunch, and called EMS from the restaurant” “feels slight chest pressure” “ongoing cough, that has worsened over the past few days” “always shortness of breath at night” “edema in legs is constant”

NKDA

Meds: Lasix, Ramipril, Atorvastatin, Spiriva, Ventolin (No recent changes, still taking them regularly)

PMH: HTN, dyslipidemia, COPD, CHF

Last meal was few hours ago

FULL CODE

**Initial conditions:**

Critical look: The patient is sitting up in bed, with 3-4 word dyspnea, using accessory muscles to breathe, GCS 15, alert and oriented to person/place/time.

Primary Survey:

Airway: pt is speaking with 3-4 word dyspnea with a normal voice, not drooling, no trismus. Appears to be protecting their airway

Breathing: Reduced air entry at bases. Crackles throughout.

Circulation: hypertensive, heart sounds irregular and rapid without murmur or gallop, neck veins difficult to make out due to thick neck, mild peripheral edema, peripheral pulses are palpable

Disability: Pupils 3mm and reactive, GCS 15

Exposure: afebrile, no skin lesions or signs of trauma, non-tender abdomen, no ascites.

Secondary Survey:

- HEENT: no cervical lymphadenopathy, neck supple

- JVP: difficult to make out due to thick neck

- Resp: as above

- CVS: as above

- Abdo: soft, non-tender, thin, no masses, no ascites

- Neuro: no localizing signs

Moulage: mild peripheral edema

POCUS conditions:

B-lines

good lung slide

IVC swollen and >2cm with no change with respiration

no pericardial effusion

no abdominal aortic aneurysm

no free fluid in the abdomen

LVH/mild decreased LV motility/EF on PLA/PSA/apical 4 chamber

| **Scenario States, Modifiers and Triggers** | | | |
| --- | --- | --- | --- |
| Patient State | Patient Status | Learner Actions, Modifiers & Triggers to Move to Next State | |
| **1. Baseline State**  Rhythm: afib  HR: 110 /min  BP: 165/95  RR: 35 /min  O_2_SAT: 89% 4L NP  T: 37.5^o^C tympanic  BG 6.2 mmol/L | GCS fluctuates from 9-12  Two to three word sentences, only ℅ SOB | Learner Actions  ☐ IV/O2/monitor  ☐ NRB to 99%  ☐ Primary survey  ☐ Secondary survey  ☐ TL verbalizes concern for resp failure due to PNA  ☐ Call for US  ☐ Discussion with TL if disagreement with plan using “CUS”  ☐ IV antibiotics | TL Actions  TL specifically anchors on the dx of pneumonia and calls for RSI. Will say that priority is ABC (ie RSI first) and ECG/CXR will delay care and cause harm    Modifiers  if large volume fluid given or at 3min →  **2. Worsening Hypoxia**    Triggers  -if students question TL direction, TL pushes back once. If met with “CUS” and/or respectful language will hesitantly engage with team only once |
| **2. Worsening Hypoxia**  Rhythm: afib  HR: 123 /min  BP: 170/99  RR: 39 /min  O_2_SAT: 89% NRB  T: 37.5^o^C oral | Patient’s GCS unchanged  Partner arrives to give collateral - main concern was dyspnea | Learner Actions  ☐ Team questions ddx using CUS  ☐ calls RT for help; will be unavailable  ☐ Team to prompt leader to order CXR/ECG/BW  ☐ Considers CHF mgmt.: NTG, BiPAP  ☐ Considers COPD: Ventolin and Atrovent nebs/MDI  ☐ Considers PE – TL discusses benefit of thrombolytics | TL Actions  TL continues to anchor on pneumonia  TL agrees to call RT if suggested by team  TL calls for IVF bolus  TL has no POCUS training  TL directs team to call medicine without any investigations    Modifiers  Patient’s GCS continues to deteriorate  → **3. Decreased LOC**  At 6 minutes → **3. Decreased LOC**    Triggers  -when medicine called, upset that no work up has been done and states will need to call back once preliminary w/u done |
| **3. Decreased LOC**  Rhythm: afib  HR: 132 /min  BP: 159/66  RR: 26 /min  O_2_SAT: 87% NRB  T: 37.5^o^C oral | Patient’s GCS goes to 9-10  Responds to painful stimuli only, starting to get tired and lower RR | Learner Actions  ☐ team to recognize decreasing LOC and notify TL (otherwise TL does not notice)  ☐ Discuss vasopressors if not given – TL ignores recommendation  ☐ Team member to perform POCUS  ☐ Call for ECG/CXR if not done  ☐ Continue to discuss ddx (CHF, PNA, A fib, COPD, etc) | TL Actions  TL continues to push for RSI despite not maximizing hemodynamics due to GCS change  If team members are having side conversations, TL gets defensive and tries to stop them    Triggers  -If vasopressors started → **6. Resolution**  Otherwise, at 8min → **ICU intervenes to end case** |

**Additional Info/Labs/Imaging:**

Hg 89 (previous 90)

WBC 22

Plt 120

Lactate 4.5

Trop 75

Na 130

K 4.5

Cl 101

AST 40

ALT 37

Bili 7

Amylase 30

VBG 7.01/CO2 55/O2 90

BNP pending

Dimer pending

**
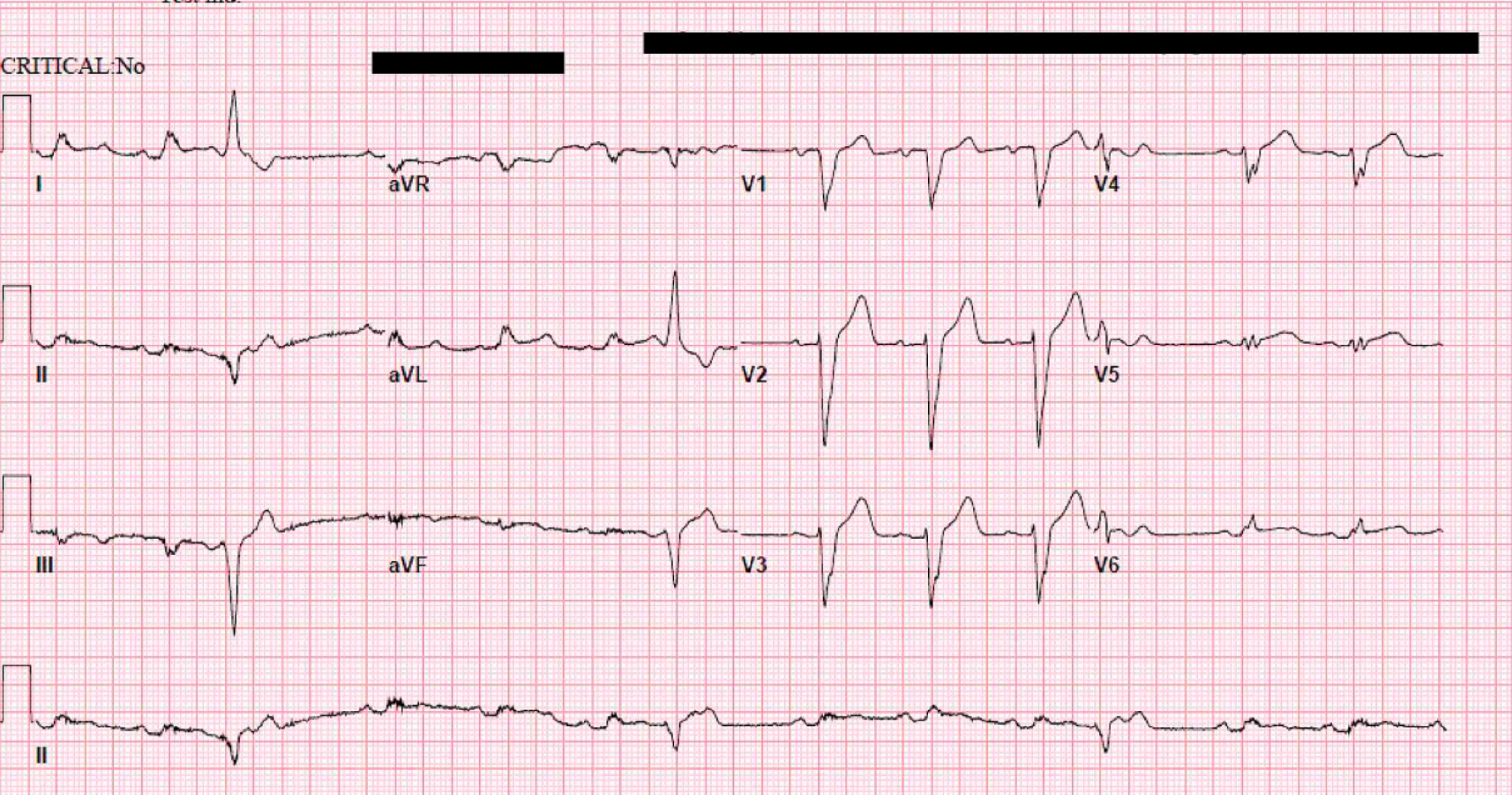
**

**
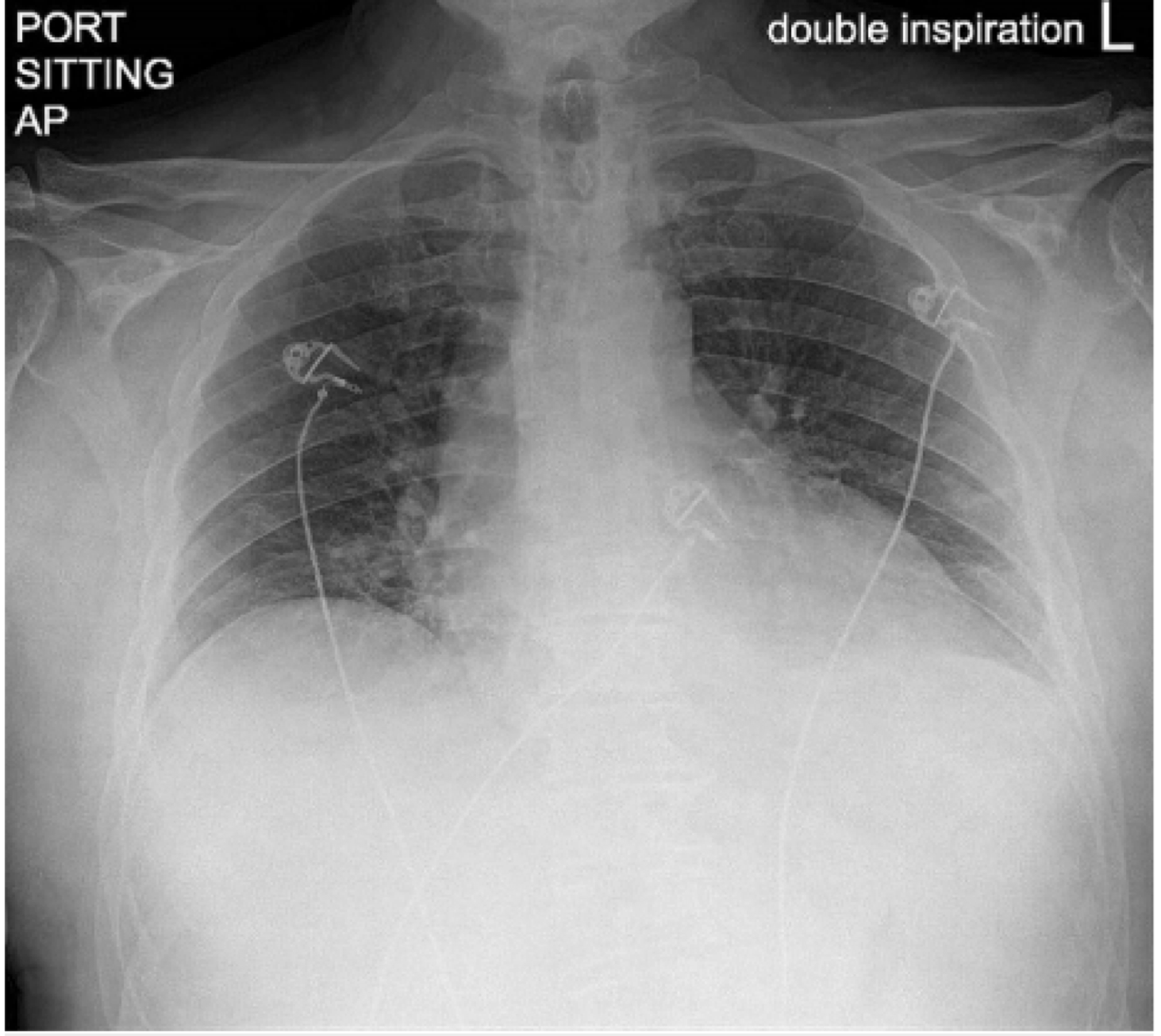
**

**Suggested debriefing/discussion points:**

Introduction

We will first go around and ask each of you how you feel coming out of that case, just your feelings.

This case was deliberately designed to have uncertainty. [Participant 1] and [Participant 2] are embedded participants, so they knew the case in advance and were deliberately leading it in a way that would create discomfort. The purpose of the simulation was to talk about unconscious bias and how that might impact our role as leaders. This is a very hard thing we’re asking you to discuss. We are asking you to be very vulnerable. We know that implicit biases are things that we all harbour and that they’re not intentional, nor do they reflect what kind of person you are. Now knowing that, who can give us a one-line recap of the case(s)?

Suggested opening questions/comments:

1. This was a case of a patient with undifferentiated shock, with multiple possible diagnoses and diagnostic uncertainty. How did it feel when the TL anchored on pneumonia?
2. If you disagreed with the TL’s differential diagnosis and management, how did you address it? How will you address it in the future?
   1. If we find ourselves in a situation where we feel like there are different mental models between the TL and team, what are some strategies we can use to address this in real-time?
3. If you noticed that ACLS was being run incorrectly, how would you address this?
4. The TL had some knowledge gaps with POCUS and ACLS, yet they seemed resistant to team member suggestions. Why do you think that is? Are there systemic reasons or sociocultural factors that you can think of?
5. When addressing the TL, did you use your CUS words? Why/Why not?
6. In our case, was there anything in their (embedded TLs) leadership styles, or the way they stood, or their body language that might have made your interactions different between them?
7. The thing about unconscious bias that’s so difficult, and why we wanted to talk about it - is because it is unconscious. None of you would choose to act in a way that influenced your decisions based off these implicit things, but it does happen. So you want to: 1. Make it conscious and be aware of it 2. Think about what we can do in real life to address it and act on it, which can be challenging.
   1. Now, is unconscious bias something that any of you were aware of before the sim ? Can any of you discuss some of the implicit biases we all hold?
      1. Have you personally ever felt like there might have been some bias at play because of the way that you present yourself to other people? Whether you look older, or younger, or have a different skin colour, the level of training you’re at, etc.
   2. When witnessing bias between colleagues and/or patients, do you know of any tools to help you address that implicit bias they may not be aware of?
8. What if a resident was the victim of bias? How might you address it with them?
   1. We could even say something like ” I observed that you were shut down multiple times when you were trying to lead that resuscitation, and to me, that felt very uncomfortable because I was worried it was based on your [height]. How did that feel for you? “
9. If you wanted to address a witnessed microaggression/bias, and not being the person experiencing it, you may want to ask your colleague, “can I address this on your behalf?”
10. If you’re someone who does command authority and command respect, automatically by virtue of whatever trait, how are you going to be safe as a new staff by creating space in resuscitation for people to speak up? What will you do?
11. Though this is a case about unconscious bias and implicit bias, it can tie into experiencing microaggressions. Can anyone give me an example of what this means ? Have you ever witnessed or experienced a microaggression?
12. We can’t run them at the same time, but knowing that this case is about implicit bias, could you think of some moments that happened that may have been different because [Participant 1] was leading it versus [Participant 2]?

**TIPS TO RESPONDING TO MICROAGGRESSIONS AND BIAS**

*Adapted from: Goodman, D. (2011). Promoting Diversity and Social Justice: Educating People from Privileged Groups. New York: Routledge. Excerpt available at www.dianegoodman.com Diane J. Goodman, Ed.D. www.dianegoodman.com*

**Restate or rephrase**

“I think I heard you saying ___________(paraphrase their comments). Is that correct”

**Ask for clarification or more information**

“Could you say more about what you mean by that ?”

“How have you come to think that?”

**Acknowledge the feelings behind the statement – express empathy and compassion.**

“It sounds like you’re really frustrated/nervous/angry….”

“I can understand that you’re upset when you feel disrespected.”

**Separate intent from impact**

“I know you didn’t realize this, but your _____________(comment/behavior) was hurtful/offensive because______________. Instead, you could _______________ (different language or behavior).

**Share your own process**

“I noticed you__________(comment/behavior). I used to do/say that too, but then I learned ______________.”

**Express your feelings**

“when you _______________(comment/behavior), I felt _____________ (feeling) and I would like you to _______________.”

**Challenge the stereotype –** give information, share your own experience and/or offer alternative perspectives.

“actually, in my experience __________________” “Another way to look at it is ______________________”

“I think that’s a stereotype. I’ve learned that ________________”

**Appeal to values and principles**

“I know you really care about ___________________. Acting in this way really undermines those intentions.”

**Promote empathy** – ask how they would feel if someone said something like that about their group/friend/partner/child

“I know you don’t like the stereotypes about __________ (their group), how do you think he feels when he hears those things about his group?”

**Point out what they have in common with the other person**

“I’m tired of hearing your Muslim jokes. Do you know he’s also studying __________and likes to ___________? You may want to talk with him about that. You actually have a lot in common.”

**What’s in it for them** – explain why diversity or that individual/group can be helpful/valuable

“I know you’re not comfortable with_________ but they can help us reach out to /better serve other patients/groups/communities

**Remind them of the rules or policies**

“that behavior is against our code of conduct and could really get you in trouble.”

**Instructor Notes/Background information:**

*The learners should formulate a differential diagnosis for shock and use the tools at their disposal to determine the cause and initiate treatments.*

This case was written to allow for uncertainty in the diagnosis. Learners should work through a ddx using bedside tools, while managing the patient’s impending hemodynamic collapse. The TL will have anchored early on a diagnosis, and in the course of managing it, may come off as being “difficult”. We hope that the debrief will allow participants to recognize gender differences in leading resuscitations and discuss the systemic factors, microaggressions, and discrimination different genders may experience.

In medical education, the most common form of abuse reported by resident physicians is gender discrimination, with a higher prevalence of discrimination and sexual harassment experienced by female than male trainees (Fnais et al., 2014). Those experiencing sexual harassment and discrimination are more likely to have decreased professional and personal satisfaction, decreased academic productivity, and are more likely to consider leaving their specialty (Fnais et al., 2014).

In Canada, equal gender representation has not yet been achieved in emergency medicine (EM); only 30.6% of emergency physicians identify as female (CMA 2019). Women EM residents have been found to experience more sexual harassment and unfair treatment due to gender than men and, consequently, were more likely to question entering the EM specialty (McNamara, Whitley, Sanders, Andrew, & Force, 1995). Comments on evaluations about male residents have been found to be more positive than those about female residents (Brucker et al., 2019; Galvin, Parlier, Martino, Scott, & Buys, 2015). In EM, nursing evaluations of residents showed that females were evaluated lower in work ethic and ability than males despite similar examination scores and milestone attainments (Brucker et al., 2019). Additionally, male residents received higher nationally standardized milestone attainments in all competencies than females with a gap equal to three months of additional training by graduation (Dayal, O’Connor, Qadri, & Arora, 2017). Qualitative analysis of EM milestone evaluation comments revealed that the “ideal” EM resident possesses stereotypically male behaviours Furthermore, they found that male residents received more consistent feedback whereas female residents received discordant feedback, especially regarding autonomy and assertiveness (Mueller et al., 2017).

Unconscious or implicit bias refers to attitudes or stereotypes about certain groups (eg. women, racial minorities, 2SLGBTQIA+ people) that unconsciously affect one’s understanding, actions and decisions. Sex and racial biases, in particular unconscious ones, often manifest in the form of microaggressions. Microaggressions were originally described by Pierce et al and can be defined as “subtle, stunning, often automatic, and non-verbal exchanges which are ‘put downs’ of blacks” and other minorities. Several studies examining how microaggressions affect women and those underrepresented in medicine. In particular, these studies have linked microaggressions to the development of anxiety, depression and even hypertension (6, 8). The first step in addressing implicit biases and microaggressions is to recognize that they exist. One study (1) discusses how we must acknowledge, explore and address the experiences of disrespect. Whether this is done through workshops, teaching conferences or other educational modules, fostering open discussion is imperative. The second step in addressing unconscious or conscious bias, is to identify strategies to counteract it. One such strategy involves developing frameworks to respond to microaggressions directly in a productive and respectful way (6). We hypothesize that simulation can be used as a modality to teach about recognition of these biases and learn tools to address microaggressions at a systemic level.

**References**

Brucker, K., Whitaker, N., Morgan, Z. S., Pettit, K., Thinnes, E., Banta, A. M., & Palmer, M. M. (2019). Exploring gender bias in nursing evaluations of emergency medicine residents. *Academic Emergency Medicine, 26*(11), 1266-1272.

Canadian Medical Association (CMA) (2018). *Addressing gender equity and diversity in in Canada’s medical profession: a review.* Equity and Diversity in Medicine. https://www.cma.ca/sites/default/files/pdf/Ethics/report-2018-equity-diversity-medicine-e.pdf.

Canadian Medical Association (CMA) (2019). *Number and percent distribution of physicians by specialty and sex, Canada 2018*. https://www.cma.ca/sites/default/files/2019-03/2018-06-spec-sex.pdf.

Dayal, A., O’Connor, D. M., Qadri, U., & Arora, V. M. (2017). Comparison of male vs female resident milestone evaluations by faculty during emergency medicine residency training. *JAMA internal medicine, 177*(5), 651-657.

Fnais, N., Soobiah, C., Chen, M. H., Lillie, E., Perrier, L., Tashkhandi, M., . . . Tricco, A. C. (2014). Harassment and discrimination in medical training: a systematic review and meta-analysis. *Academic Medicine, 89*(5), 817-827.

Galvin, S. L., Parlier, A. B., Martino, E., Scott, K. R., & Buys, E. (2015). Gender bias in nurse evaluations of residents in obstetrics and gynecology. *Obstetrics & Gynecology, 126*, 7S-12S.

Gerull, K. M., Loe, M., Seiler, K., McAllister, J., & Salles, A. (2019). Assessing gender bias in qualitative evaluations of surgical residents. *The American Journal of Surgery, 217*(2), 306-313.

Klein, R., Julian, K. A., Snyder, E. D., Koch, J., Ufere, N. N., Volerman, A., . . . Palamara, K. (2019). Gender bias in resident assessment in graduate medical education: review of the literature. *Journal of general internal medicine, 34*(5), 712-719.

McNamara, R. M., Whitley, T. W., Sanders, A. B., Andrew, L. B., & Force, F. t. S. I. s. S. T. (1995). The extent and effects of abuse and harassment of emergency medicine residents. *Academic Emergency Medicine, 2*(4), 293-301.

Mueller, A. S., Jenkins, T. M., Osborne, M., Dayal, A., O'Connor, D. M., & Arora, V. M. (2017). Gender differences in attending physicians' feedback to residents: a qualitative analysis. *Journal of graduate medical education, 9*(5), 577.
