# Supplementary material for: Simulation as an educational tool to teach emergency medicine residents about unconscious bias
Source: CJEM. 2024 Mar 26;26(6):395–8. doi: 10.1007/s43678-024-00679-3 (PMC11189339; doi:10.1007/s43678-024-00679-3)
Supplement: Supplementary file 2 — Supplementary file2 (DOCX 17 KB) [file 43678_2024_679_MOESM2_ESM.docx]

**APPENDIX B -** *DEFINITIONS*

ALLY

A person who works to end a form of oppression that gives them privilege(s). Allies listen to, and are guided by, communities and individuals affected by oppression. Forms of oppression include: able-ism, ageism, audism, classism, biphobia, homophobia, transphobia, racism, sexism, and others [1a].

BINARY GENDER

Gender binary, a system that classifies [sex](https://www.britannica.com/science/sex) and [gender](https://www.britannica.com/topic/gender-identity) into a pair of opposites, often [imposed](https://www.britannica.com/dictionary/imposed) by [culture](https://www.britannica.com/topic/culture), [religion](https://www.britannica.com/topic/religion), or other societal pressures. Within the [gender](https://www.britannica.com/topic/gender-grammar) binary system, all of the [human](https://www.britannica.com/topic/human-being) population fits into one of two genders: man or woman [2a].

CIS/CISGENDER

Cisgender is used to explain the phenomena where a person's gender identity is in line with or "matches" the sex they were assigned at birth. Cis can also be used as a prefix to an assortment of words to refer to the alignment of gender identity and the assigned at birth sex status including; cisnormativity, cissexual, cisgender, cis male, and cis female [1a].

DISCRIMINATION

Any form of unequal treatment based on a ground protected by human rights legislation, that results in disadvantage, whether imposing extra burdens or denying benefits. Discrimination can be intentional or unintentional; and it may occur at an individual or systemic level. It may include direct actions or more subtle aspects of rules, practices and procedures that limit or prevent access to opportunities, benefits, or advantages that are available to others [1a].

EDIIA

Equity, Diversity, Inclusivity, Indigeneity, Accessibility

### GENDER IDENTITY

A person's internal and individual experience of gender. It is a person's sense of being a woman, a man, both, neither, or anywhere along the gender spectrum. A person's gender identity may be the same as or different from their birth-assigned sex. A person's gender identity is fundamentally different from and not related to their sexual orientation [1a].

### GENDERQUEER/GENDER NON-CONFORMIN/GENDER VARIANT

### Individuals who do not follow gender stereotypes based on the sex they were assigned at birth. They may identify and express themselves as “feminine men” or “masculine women” or as androgynous, outside of the categories “boy/man” and “girl/woman.” People who are gender non-conforming may or may not identify as trans [1a].

INTERSECTIONALITY

When two or more oppressions overlap in the experiences of an indi­vidual or group, creating interconnected barriers and complex forms of discrimination that can be insidious, covert and compounded [1a].

MICROAGGRESSIONS

Microaggressions are everyday verbal, nonverbal, and environmental slights, snubs, or insults -- whether intentional or unintentional -- that communicate hostile, derogatory, or negative messages to individuals based solely upon their marginalized group membership*. Microaggressions repeat or affirm stereotypes about a minority group, and they tend to minimize the existence of discrimination or bias, intentional or not [3a].

### TRANS/TRANSGENDER

### Umbrella terms that describe people with diverse gender identities and gender expressions that do not conform to stereotypical ideas about what it means to be a girl/woman or boy/man in society. “Trans” can mean transcending beyond, existing between, or crossing over the gender spectrum. It includes but is not limited to people who identify as transgender, transsexual, cross-dressers or gender non-conforming (gender variant or gender-queer).

Trans identities include people whose gender identity is different from the gender associated with their birth-assigned sex. Trans people may or may not undergo medically sup­portive treatments, such as hormone therapy and a range of surgical procedures, to align their bodies with their internally felt gender identity [1a].

UNCONSCIOUS BIAS

Unconscious bias (or implicit bias) is often defined as prejudice or unsupported judgments in favor of or against one thing, person, or group as compared to another, in a way that is usually considered unfair. Many researchers suggest that unconscious bias occurs automatically as the brain makes quick judgments based on past experiences and background. As a result of unconscious biases, certain people benefit and other people are penalized. In contrast, deliberate prejudices are defined as conscious bias (or explicit bias). Although we all have biases, many unconscious biases tend to be exhibited toward minority groups based on factors such as class, gender, sexual orientation, race, ethnicity, nationality, religious beliefs, age, disability and more [4a].

**References:**

1a. <https://www.rainbowhealthontario.ca/news-publications/glossary/#gender>

2a. https://www.britannica.com/topic/gender-binary

3a. <https://equity.ucla.edu/wp-content/uploads/2016/06/DiversityintheClassroom2014Web.pdf>

4a. <https://www.vanderbilt.edu/diversity/unconscious-bias/#:~:text=Unconscious%20bias%20(or%20implicit%20bias,that%20is%20usually%20considered%20unfair>.
